# Ophthalmomyiasis Caused by *Chrysomya* *bezziana* after Periocular Carcinoma

**DOI:** 10.3201/eid2511.181706

**Published:** 2019-11

**Authors:** Reza Nabie, Adel Spotin, Bayan Poormohammad

**Affiliations:** Tabriz University of Medical Sciences, Tabriz, Iran

**Keywords:** ophthalmomyiasis, squamous cell carcinoma, Chrysomya bezziana, eyes, parasites, screwworm fly, screwworm, Iran

## Abstract

We treated a homeless man in Iran with a history of squamous cell carcinoma who had ophthalmomyiasis caused by *Chrysomya* *bezziana* parasites. This case highlights a much-neglected condition and describes measures to prevent it.

Ophthalmomyiasis is principally manifested as orbital myiasis, ophthalmomyiasis external, and ophthalmomyiasis interna (*1**,**2*). *Chrysomya* *bezziana* (screwworm) has been implicated in cancer-associated myiasis of the skin, larynx, face, and breast (*3**–**5*). Ophthalmomyiasis is uncommon but becomes significant in debilitated and compromised patients.

In December 2017, a 75-year-old homeless man sought care at the University Hospital of Nikookari Eye Center (Tabriz, northwestern Iran). His medical history included a surgery for left-side periocular squamous cell carcinoma ≈15 years earlier. After 2 years, the patient observed recurrence of the squamous cell carcinoma, but he did not seek further evaluation and treatment. He reported loss of sight since 2015 because of the tumor extension into the orbit and globe of his eye. He had intermittent pain.

On examination, his systemic findings were unremarkable. Orbital and periocular examination revealed extensive tissue necrosis and extension to the eyelids, eyebrow, and orbit (Figure, panel A). The globe seemed to be totally invaded and necrotized by the tumor. A computed tomography scan of the area showed huge invasion and necrosis of all cavities of the orbit, including the globe and bone absorption of the superotemporal area of orbit; we further suspected extension of the tumor to the maxillary and ethmoidal sinuses (Figure, panel B). We also found different sizes of live larvae inside the necrotic tissue of orbit and periorbital skin (Figure, panel C; Video). 

**Figure:**
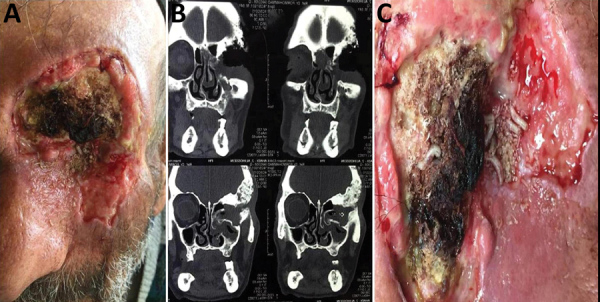
A 75-year-old man with ophthalmomyiasis after periocular squamous cell carcinoma, Iran. A) Extensive tissue necrosis and the extension of the eyelids, eyebrow, and orbit. B) Computed tomography scan showing huge invasion and necrosis of all cavities of the orbit. C) Live screwworm larvae (*Chrysomya* *bezziana*) inside the necrotic tissue of the orbit.

**Video:** Ophthalmomyiasis caused by live larvae of *Chrysomya bezziana* in 75-year-old man, Iran.

Our sequencing and phylogenetic analyses of cytochrome b gene showed that isolated larvae (GenBank accession no. MN158142) were confirmed as *C. bezziana* with 100% identity (query coverage: 100%) isolated from livestock in Oman. We planned to admit the patient for further management, but he denied the admission and accepted only irrigation of the site with normal saline. After irrigation that removed only the superficial larvae, he left the hospital and never returned for further treatment.

This case highlights a much-neglected squamous cell carcinoma of the periorbital region with orbital invasion necrosis of globe and orbital soft tissue and massive and extensive larvae infestation caused by the *C. bezziana* screwworm. To prevent myiasis cases such as the one we describe, healthcare providers should emphasize general cleanliness of surroundings, maintenance of good personal hygiene, provision of basic sanitation, and health education. 

## References

[1] Khataminia G, Aghajanzadeh R, Vazirianzadeh B, Rahdar M. Orbital myiasis. J Ophthalmic Vis Res. 2011;6:199–203. 22454736 PMC3306096

[2] Sigauke E, Beebe WE, Gander RM, Cavuoti D, Southern PM. Case report: ophthalmomyiasis externa in Dallas County, Texas. Am J Trop Med Hyg. 2003;68:46–7. 10.4269/ajtmh.2003.68.46 12556147

[3] Hawayek LH, Mutasim DF. Myiasis in a giant squamous cell carcinoma. J Am Acad Dermatol. 2006;54:740–1. 10.1016/j.jaad.2005.07.012 16546611

[4] Rubio C, Ladrón de Guevara C, Martín MA, Campos L, Quesada A, Casado M. [Cutaneous myiasis over tumor-lesions: presentation of three cases] [in Spanish]. Actas Dermosifiliogr. 2006;97:39–42. 10.1016/S0001-7310(06)73346-4 16540050

[5] Kwong A, Yiu WK, Chow LW, Wong S. *Chrysomya bezziana*: a rare infestation of the breast. Breast J. 2007;13:297–301. 10.1111/j.1524-4741.2007.00426.x 17461907

