# Supplementary figures and images for: Ophthalmomyiasis Caused by Chrysomya bezziana after Periocular Carcinoma
Source: Emerg Infect Dis. 2019 Nov;25(11):2123–4. doi: 10.3201/eid2511.181706 (PMC6812677; doi:10.3201/eid2511.181706)

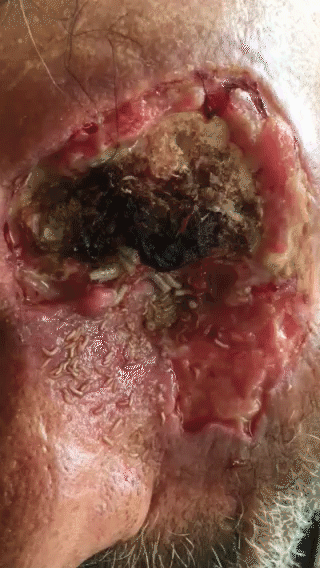

Supplement: Supplementary file 1 [file 18-1706-vid1.gif]
